# Loss of DIAPH3, a Formin Family Protein, Leads to Cytokinetic Failure Only under High Temperature Conditions in Mouse FM3A Cells

**DOI:** 10.3390/ijms21228493

**Published:** 2020-11-11

**Authors:** Hiroki Kazama, Shu-ichiro Kashiwaba, Sayaka Ishii, Keiko Yoshida, Yuta Yatsuo, Takuma Naraoka, Masashi Fukuoka, Yasufumi Murakami

**Affiliations:** Department of Biological Science and Technology, Faculty of Industrial Science and Technology, Tokyo University of Science, Tokyo 125-8585, Japan; 8319523@ed.tus.ac.jp (H.K.); kashiwaba@omr.co.jp (S.-i.K.); 8317605@ed.tus.ac.jp (S.I.); 8318569@ed.tus.ac.jp (K.Y.); 8312092@ed.tus.ac.jp (Y.Y.); 8313074@ed.tus.ac.jp (T.N.); masashifukuoka@rs.tus.ac.jp (M.F.)

**Keywords:** cell division, mitosis, temperature-sensitive mutants, formin family proteins, *Diaph3*

## Abstract

Cell division is essential for the maintenance of life and involves chromosome segregation and subsequent cytokinesis. The processes are tightly regulated at both the spatial and temporal level by various genes, and failures in this regulation are associated with oncogenesis. Here, we investigated the gene responsible for defects in cell division by using murine temperature-sensitive (ts) mutant strains, tsFT101 and tsFT50 cells. The ts mutants normally grow in a low temperature environment (32 °C) but fail to divide in a high temperature environment (39 °C). Exome sequencing and over-expression analyses identified *Diaph3*, a member of the formin family, as the cause of the temperature sensitivity observed in tsFT101 and tsFT50 cells. Interestingly, *Diaph3* knockout cells showed abnormality in cytokinesis at 39 °C, and the phenotype was rescued by re-expression of *Diaph3* WT, but not *Diaph1* and *Diaph2*, other members of the formin family. Furthermore, *Diaph3* knockout cells cultured at 39 °C showed a significant increase in the level of acetylated α-tubulin, an index of stabilized microtubules, and the level was reduced by *Diaph3* expression. These results suggest that *Diaph3* is required for cytokinesis only under high temperature conditions. Therefore, our study provides a new insight into the mechanisms by which regulatory factors of cell division function in a temperature-dependent manner.

## 1. Introduction

Cytokinesis occurs after partitioning the duplicated chromosomes into two nuclei, and these nuclei were separated into daughter cells by the cleavage furrow (review articles [1,2]). In animal cells, the cleavage site was firstly specified by the centralspindlin complex and chromosome passenger complex (CPC). The complexes activate downstream signaling proteins including the key guanine nucleotide exchange factor Rho-GEF named Ect-2, Rho family GTPases, and proteins that act locally to assemble a contractile ring, which is assembled around the equator of the dividing cells. The contractile ring was composed of actin filaments and myosin-II and assembled as shown below. The primary signal to assemble the contractile ring comes from Ect-2 that activates the Rho-GTPase. The active Rho-GTPase drives the contractile ring assembly in animal cells by activating Rho-kinase (ROCK) to phosphorylate the regulatory light chains of myosin-II. The active Rho-GTPase also drives the assemble of actin filaments into the contractile ring by activating formins, which have instrumental roles in controlling rearrangements of the actin cytoskeleton. Finally, interactions of myosin-II with actin filaments produce force to assemble and then constrict the contractile ring to form the cleavage furrow. Contractile rings disassemble as they constrict, resulting in the completion of cytokinesis.

Defects in the regulatory mechanisms of cytokinesis lead to the increase or decrease in the number of chromosomes that are passed on to daughter cells, leading to chromosome instability [3]. This can alter the expression of a large number of genes and function of proteins, ultimately promoting oncogenesis of cells and their malignant transformation [4]. Additionally, abnormalities in chromosome distribution during meiosis lead to trisomy syndromes such as Down’s syndrome, in which three copies of chromosome 21 are present, and Klinefelter’s disease, in which the male sex chromosome type is XXY [5,6]. Therefore, the detailed understanding of the mechanisms of mammalian cell division is important from both a basic biological as well as a medical point of view, as it may lead to a better insight into the pathogenesis of these diseases and their treatment applications. The use of RNAi and gene knockout, for example, may be an analytical approach to uncover the details of cell division mechanisms. However, if the gene of interest is essential for cell division, it is difficult to analyze it since cells cannot proliferate.

Temperature-sensitive (ts) mutant strains are useful for analyzing the genes essential for cell division mechanisms [7]. In general, it is believed that missense mutations in genes are responsible for the temperature sensitivity of ts mutant strains under high temperature conditions. Such missense mutations alter codons and trigger amino acid substitutions in gene products. Under high temperature conditions, the proteins encoded by the genes become unstable in their conformation and are unable to function normally; consequently, they are sensitive to temperature. The ts mutants are well used for studies in yeast. Additionally, in mammalian studies, ts mutant strains exhibiting phenotypes such as abnormalities in cell division at a restrictive temperature (39 °C) have been generated. For example, tsFT20 cells fail to synthesize DNA at 39 °C due to a mutation in DNA polymerase α, and tsFT210 cells are arrested at the G2 phase at 39 °C due to a mutation in *Cdc2*. The ts mutant strains have greatly contributed to the study of mammalian cell cycle [8,9,10]. In addition to these strains, there are many other ts mutants (e.g., tsFT101, tsFT50) for which the responsible gene has not been identified until date. The ts mutant cell lines were established by treating a mouse breast cancer FM3A cell line with N-methyl-N′-nitro-N-nitrosoguanidine (MNNG), resulting in random mutations in its chromosomes [11]. In addition to missense mutations that result in protein dysfunction only at the restrictive temperature, the MNNG treatment may give rise to mutations (e.g., frameshift and nonsense mutations) that prevent the production of the target protein even at the permissive temperature. Therefore, if we can identify mutations in the gene responsible for ts mutant strains, we may be able to identify genes that are essential for cell division only under high temperature conditions.

In this study, we focused on tsFT101 and tsFT50 cells, which show a marked temperature sensitivity among the mouse ts mutant strains for which the responsible gene has not been identified. Exome analysis and gene transfer experiments suggested that *diaphanous related formin 3* (*Diaph3)*, which was observed to be mutated in both cells, was the responsible gene for the thermosensitivity. Surprisingly, tsFT50 cells grew at a permissive temperature (32 °C) despite the absence of *Diaph3* expression, suggesting that *Diaph3* is necessary for cell growth under high temperature conditions. Furthermore, although *Diaph3* knockout in the parental FM3A cells did not affect cell division and growth at 32 °C, the knockout cells showed temperature sensitivity with multinucleation and a decrease in cell growth at 39 °C. *Diaph3*, a member of formin family, is known to function in actin fiber formation via its formin homology 2 (FH2) domain [12]. The FH2 domain is the actin nucleating domain, and the mutations in the FH2 domain lead to a decrease in the actin polymerization activity [13]. However, not all mutations in the FH2 domain affect the temperature-dependent cytokinesis defect in *Diaph3* KO cells, suggesting that *Diaph3* has some functions other than the actin nucleating function. Finally, our analysis revealed that *Diaph3* is involved in the destabilization of microtubule in cytokinesis at 39 °C. The results suggest that *Diaph3* might regulate cytokinesis only under high temperature conditions via controlling the stability of microtubule directly or indirectly. Therefore, our study will shed light on the new regulatory mechanism through a temperature-dependent factor.

## 2. Results

### 2.1. Diaph3 is the Gene Responsible for Temperature Sensitivity of tsFT101 Cells under High Temperature Conditions

**Figure 1:**
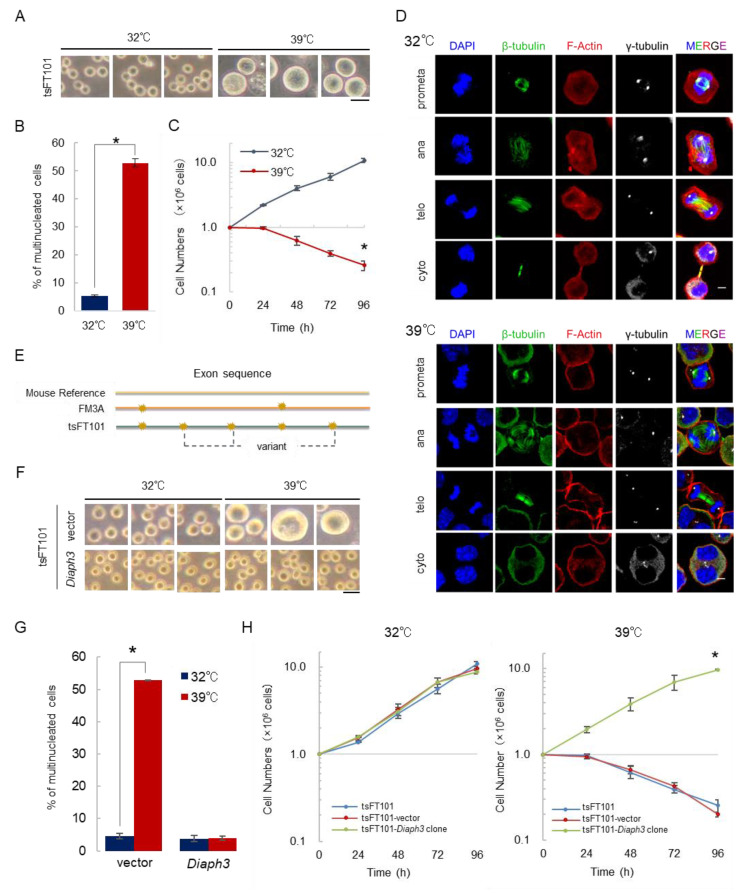
*Diaph3* is the gene responsible for temperature sensitivity of tsFT101 cells under high temperature conditions. (**A**) Cell images: tsFT101 cells were cultured at 32 or 39 °C for 96 h under 5% CO_2_. The scale bar indicates 50 µm. (**B**) The tsFT101 cells were incubated at 32 and 39 °C for 24 h, and DAPI staining was performed. More than 300 cells were measured and the percentage of multinucleated cells was calculated; (error bars: ±SEM); * *p* < 0.001 (Student’s t-test); 32 vs. 39 °C. (**C**) Growth curve analysis of tsFT101 cells. Cells were seeded at 1.0 × 10^6^ cells/dish and incubated at 32 or 39 °C under 5% CO_2_ and collected every 24 h to measure the number of cells; (error bars: ±SEM); * *p* < 0.001 (Student’s t-test); 32 vs. 39 °C. (**D**) To observe the behavior of tsFT101 cells in cell division, they were incubated at 32 and 39 °C for 24 h and then immunostained with antibodies specific for β-tubulin (microtubules: Green) and γ-tubulin (centrosomes: White). DAPI (blue) and Phalloidin (red) were used to stain chromosomes and F-actin, respectively. The scale bar indicates 10 µm. (**E**) Schematic diagram of exome sequencing analysis. A mouse reference sequence, exon sequence of FM3A cells, and tsFT101 cells were compared to select variants present in tsFT101 cells. Yellow flashes on the diagram indicate the variants observed at the genomes of each cell line. (**F**) Cell images: The tsFT101-vector cells and tsFT101-*Diaph3* cells (bulk) were cultured at 32 or 39 °C for 96 h under 5% CO_2_. The scale bar indicates 50 µm. (**G**) Control tsFT101cells (vector) and tsFT101-*Diaph3* cells (*Diaph3*) were cultured at 32 and 39 °C for 24 h. The percentage of multinucleated cells was calculated as described in (**B**); (error bars: ±SEM); * *p* < 0.001 (Student’s t-test); 32 vs. 39 °C. (**H**) Growth curve analysis of tsFT101, tsFT101-vector, and tsFT101-*Diaph3* clone cells was performed as described in (**C**); (error bars: ±SEM); * *p* < 0.001 (Student’s t-test); tsFT101 cells (39 °C, 96 h) vs. tsFT101-*Diaph3* (39 °C, 96 h).

The ts mutant tsFT101 cells divide normally at a permissive temperature (32 °C), but show a multinucleated phenotype at a restrictive temperature (39 °C) [11,14]. To confirm the phenotypes, we first analyzed the characteristics of tsFT101 cells at 32 and 39 °C, and the cells cultured at 39 °C displayed giant sizes than those cultured at 32 °C (Figure 1A). In addition, the nuclei of the cells incubated at each temperature were stained, and the percentage of multinucleated cells was calculated. The results indicated that the percentage of multinucleated cells was significantly increased in tsFT101 cells at 39 °C, as previously reported (Figure 1B) [11,14]. The growth curve analysis at each temperature showed a rapid decrease in the number of cells at 39 °C (Figure 1C). To investigate at which stage of cell division the tsFT101 cells failed to divide, we examined cell division stages by immunofluorescence analysis. The results indicated that the cells completed the prometaphase, anaphase, and telophase at 32 and 39 °C. Although the separation of chromosomes was completed, the cells failed in cytoplasmic division only at 39 °C, resulting in multinucleation (Figure 1D). The ts mutants are thought to exhibit thermosensitivity through the introduction of mutations that cause amino acid substitutions. Such mutations destabilize protein structures at high temperatures, resulting in reduced or inactivated protein functions [9,10,15]. Therefore, we performed the exome sequencing analysis to investigate why tsFT101 cells fail in the process of cytokinesis under restrictive temperature conditions. The variants present only in tsFT101 cells were filtered by comparing the exon sequence of tsFT101 cells with that of parental FM3A cells and a mouse reference sequence (Figure 1E). We first selected four genes involved in cytokinesis (Table 1). Furthermore, we focused on *Diaph3* among the genes, since the exome analysis revealed that the *Diaph3* mutation in tsFT101 cells is the missense homozygous mutation in which I733 is replaced by asparagine (DIAPH3^I733N^) on the FH2 domain, which is the actin nucleating domain (Figure S1) [13]. Therefore, we established stable wild-type *Diaph3*-expressing tsFT101 cells (tsFT101-*Diaph3*) and examined the phenotypes. Surprisingly, the tsFT101-*Diaph3* cells did not show cell enlargement at 39 °C (Figure 1F), and the percentage of multinucleated cells was significantly decreased (Figure 1G). In addition, *Diaph3* expression led to normal cell growth at 39 °C (Figure 1H). This suggests that DIAPH3^I733N^ is sensitive to high temperature conditions due to the reduced actin polymerization activity in the cells. Thus, we stably expressed DIAPH3^I733N^ in tsFT101 cells. However, the cells did not recover from the temperature sensitivity under high temperature conditions (Figure S2). Therefore, these results suggest that *Diaph3* is the gene responsible for the temperature sensitivity of tsFT101 cells.

### 2.2. Diaph3 Is not Expressed at the Protein Level in tsFT50 Cells

**Figure 2:**
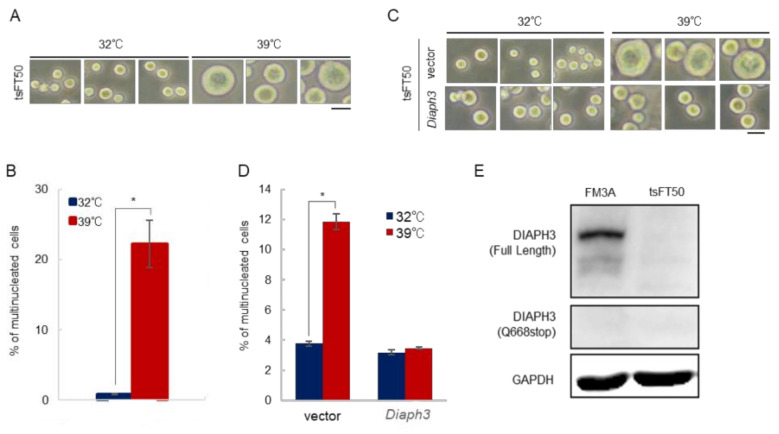
*Diaph3* is not expressed at the protein level in tsFT50 cells. (**A**) Cell images: The tsFT50 cells were cultured at 32 or 39 °C under 5% CO_2_ for 96 h. The scale bar indicates 50 µm. (**B**) The tsFT50 cells were incubated at 32 and 39 °C for 24 h and were then stained with DAPI. More than 300 cells were measured, and the percentage of the multinucleated cells was calculated; (error bars: ±SEM); * *p* < 0.01 (Student’s t-test); 32 vs. 39 °C. (**C**) Control tsFT50 cells (vector) and *Diaph3*-expressing tsFT50 cells (*Diaph3*) were cultured at 32 or 39 °C under 5% CO_2_ for 96 h. The scale bar indicates 50 µm. (**D**) Control tsFT50 cells (vector) and *Diaph3*-expressing tsFT50 cells (*Diaph3*) were cultured at 32 and 39 °C for 24 h. The percentage of multinucleated cells were calculated as described in (**B**); (error bars: ±SEM); * *p* < 0.01 (Student’s t-test); 32 vs. 39 °C. (**E**) Expression of *Diaph3* at 32 °C in FM3A and tsFT50 cells was confirmed by Western blotting. Anti-DIAPH3 antibodies recognizing residues 554–662 of DIAPH3 were used; GAPDH was used as a loading control.

Next, we studied another mammalian ts mutant strain—tsFT50. The tsFT50 cells also show a multinucleated phenotype at restrictive temperatures similar to that of tsFT101 cells. The cause of temperature sensitivity of tsFT50 cells is still unclear, and thus, we investigated the basis of this sensitivity. The tsFT50 cells cultured at 39 °C displayed giant sizes than those cultured at 32 °C, and the percentage of multinucleated cells were significantly increased (Figure 2A,B). We also examined whether tsFT50 cells could grow at 39 °C and observed a delay in cell growth at 39 °C than at 32 °C (Figure S3A). As with tsFT101 cells, we also studied the cell division of tsFT50 cells under both low and high temperature conditions to determine at which stage of cell division the cells show abnormalities. In contrast to the tsFT101 cells, a multipolar phenotype was observed in tsFT50 cells (Figure S3B). Next, we performed the exome analysis to identify the genes responsible for temperature sensitivity of tsFT50 cells and found four candidate genes involved in cell division (Table 2). Interestingly, *Diaph3*, a gene responsible for tsFT101 cells, was also found in the candidate genes. Therefore, we established stable *Diaph3*-expressing tsFT50 cells (tsFT50-*Diaph3*) to confirm whether the cells recovered from temperature sensitivity. The size of tsFT50-*Diaph3* cells cultured at 39 °C were almost the same as that of 32 °C (Figure 2C). In addition, there was no change in the percentage of multinucleated cells compared to that at 32 °C, suggesting that the cells recovered from temperature sensitivity (Figure 2D). The growth of tsFT50-*Diaph3* at 39 °C was not different from that at 32 °C (Figure S3C). Considering that tsFT50 and tsFT101 cells showed failure in cell division at different stages under high temperature conditions, there may be additional factors, other than *Diaph3,* that induce temperature sensitivity in both types of cells.

The exome analysis revealed that the *Diaph3* mutation in tsFT50 cells is a nonsense homozygous mutation in which Q668 is replaced by a stop codon (Figure S4). Therefore, we examined the expression of *Diaph3* in tsFT50 cells and found that *Diaph3* was expressed neither as a full-length protein (~134 kDa) nor as a short fragment (~76 kDa), which is thought to be obtained by the Q668* mutation (Figure 2E). We hypothesized that this was due to the instability and degradation of the protein structure as a result of its fragmentation into shorter peptide fragments. Regarding tsFT101 cells, *Diaph3* was normally expressed at 32 °C (Figure S5). Taken together, these results indicate that tsFT50 cells could divide and grow normally at 32 °C even in the absence of *Diaph3* expression. Our results also suggest that the need for *Diaph3* in cell division changes with temperature.

### 2.3. Diaph3 Knockout in FM3A Cells also Induces Temperature Sensitivity 

**Figure 3:**
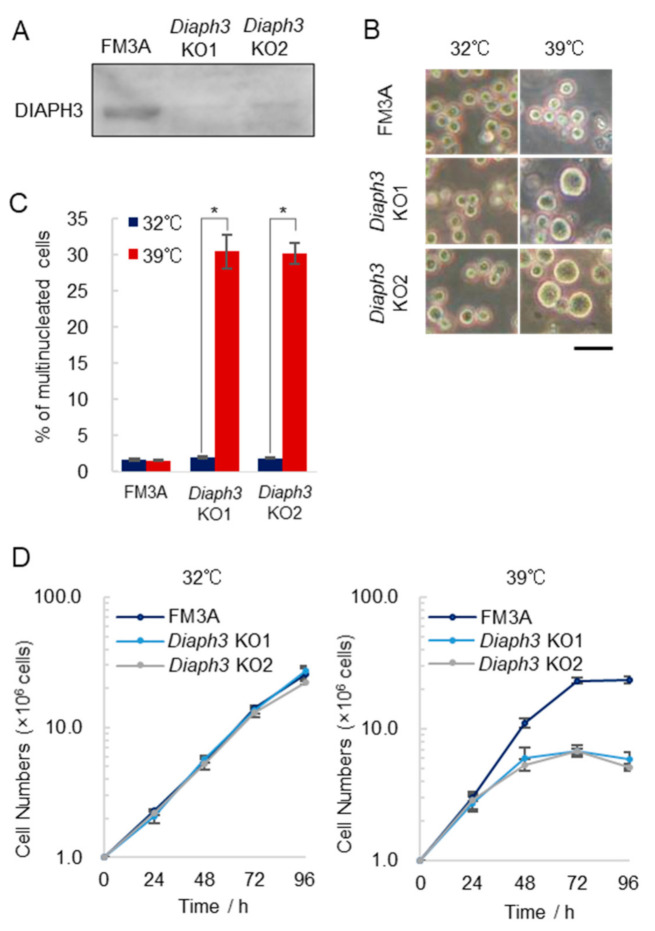
*Diaph3* knockout cells are temperature-sensitive under high temperature conditions. (**A**) *Diaph3* knockout FM3A cells (*Diaph3* KO1 and *Diaph3* KO2) were established by the CRISPR-Cas9 system, and the expression of *Diaph3* in the cells was examined by Western blotting. (**B**) Cell images: FM3A and *Diaph3* KO cells were cultured at 32 or 39 °C for 96 h under 5% CO_2_. The scale bar indicates 20 µm. (**C**) FM3A and *Diaph3* KO cells were cultured at 32 and 39 °C for 24 h and subsequently stained with DAPI. More than 300 cells were measured and the percentage of multinucleated cells was calculated; (error bars: ±SEM); * *p* < 0.001 (Dunnett’s test); 32 vs. 39 °C. (**D**) Growth curve analysis of FM3A and *Diaph3* KO cells: Cells (1.0 × 10^6^) were seeded and cultured at 32 or 39 °C under 5 % CO_2_; (error bars: ±SEM); * *p* < 0.001 (Student’s t-test); FM3A (39 °C) vs. *Diaph3* KO (39 °C).

*Diaph3* was the cause of temperature sensitivity in tsFT50 and tsFT101 cells at restrictive temperatures but was not expressed in tsFT50 cells. However, the tsFT strains were generated by introducing random mutations into the genome of FM3A cells, and mutations other than *Diaph3* were reliably introduced in both tsFT50 and tsFT101 cells. Therefore, we could not exclude the effect of other mutations on the temperature sensitivity observed in the ts mutants. To test whether *Diaph3* acts as a trigger for temperature sensitivity alone, we knocked out *Diaph3* in FM3A cells (*Diaph3* KO cells) and examined the temperature sensitivity under high temperature conditions (Figure 3A). Surprisingly, *Diaph3* KO cells were observed to occur as giant cells at 39 °C, while no giant cells were observed at 32 °C (Figure 3B). Additionally, the percentage of multinucleated cells was significantly increased at 39 compared to 32 °C (Figure 3C). Interestingly, multinucleated cells were dramatically increased at 39 but not at 37 °C (Figure S6). The ability of the cells to grow was also reduced at 39 °C, suggesting that *Diaph3* KO cells were temperature-sensitive (Figure 3D). These results indicated that the cells could grow at 32 °C in the absence of *Diaph3* but were thermosensitive at 39 °C. In other words, our results suggest that *Diaph3* is required for accurate cell division only at high temperatures such as 39 °C.

### 2.4. Overexpression of Diaph3, but Not Diaph1 and Diaph2, Rescues the Temperature Sensitivity Exhibited by Diaph3 KO Cells under High Temperature Conditions

**Figure 4:**
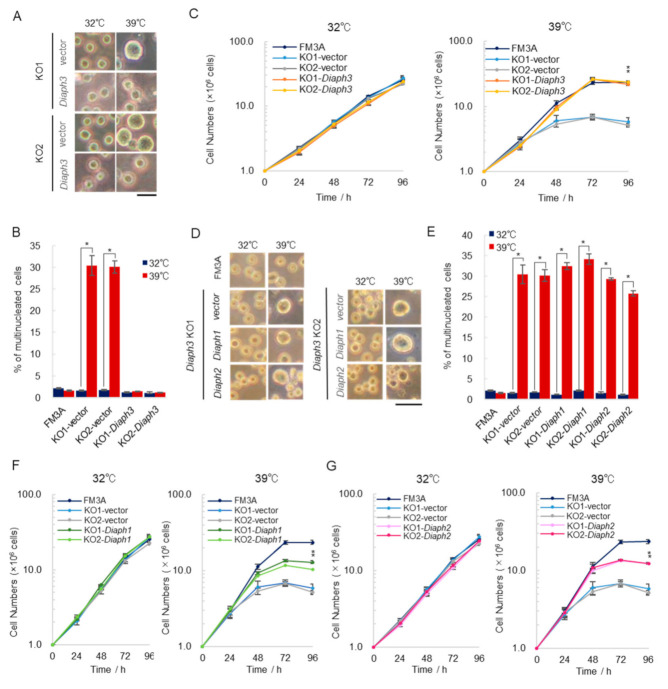
DIAPH3 is required for cell division only under high temperature conditions. (**A**) Cell images: *Diaph3* KO1 and *Diaph3* KO2 cells (KO1 and KO2) stably expressing *Diaph3* were established and KO-vector and KO-*Diaph3* cells were cultured for 96 h at 32 or 39 °C under 5% CO_2_. The scale bar indicates 20 µm. (**B**) Control *Diaph3*-KO cells (KO-vector) and *Diaph3* expressing *Diaph3*-KO cells (KO-*Diaph3*) were cultured at 32 and 39 °C for 24 h and were then subjected to DAPI staining. More than 300 cells were measured, and the percentage of multinucleated cells was calculated; (error bars: ±SEM); * *p* < 0.001 (Dunnett’s test); 32 vs. 39 °C. (**C**) Growth curve analysis of KO-vector and KO-*Diaph3* cells: Cells were seeded at 1.0 × 10^6^ cells/dish and incubated at 32 or 39 °C under 5 % CO_2_. Cells were collected every 24 h to determine the number of cells; (error bars: ±SEM); * *p* < 0.001 (Dunne tt’s test); KO-vector vs. KO-*Diaph3.* (**D**) FM3A cells, KO-vector cells, *Diaph1* expressing *Diaph3*-KO cells (KO-*Diaph1* cells), and *Diaph2* expressing *Diaph3*-KO cells (KO-*Diaph2* cells) were cultured for 96 h at 32 or 39 °C under 5% CO_2_ and photographed. The scale bar indicates 20 µm. (**E**) FM3A, KO-vector, KO-*Diaph1*, and KO-*Diaph2* cells were cultured at 32 and 39 °C for 24 h. The percentage of multinucleated cells was calculated as described in (**B**) (error bars: ±SEM); * *p* < 0.001 (Dunnett’s test); 32 vs. 39 °C. (**F**) Growth curve analysis of FM3A cells, KO-vector cells, and KO-*Diaph1* cells was performed as described in (**C**); (error bars: ±SEM); * *p* < 0.001 (Dunnett’s test); KO-vector vs. KO-*Diaph1*. (**G**) Growth curve analysis of FM3A cells, KO-vector cells, and KO-*Diaph2* cells was performed as described in (**F**).

We tested whether the stable expression of *Diaph3* in *Diaph3* KO cells could rescue them from temperature sensitivity. We cultured *Diaph3* KO-*Diaph3* (KO-*Diaph3*) cells at 32 and 39 °C, and almost no giant cells were observed at both temperatures (Figure 4A). The percentage of multinucleated cells and cell growth at 39 °C were similar to those at 32 °C (Figure 4B,C). Therefore, the re-expression of *Diaph3* restored the phenotypes at 39 °C, further suggesting that *Diaph3* is required only under high temperature conditions.

*Diaph1* and *Diaph2*, other formin family members, have similar domain structures. These two formins have similar domain structures and have been reported to show similar actin polymerization ability in vitro [16]. We hypothesized that the expression of *Diaph1* and *Diaph2* in *Diaph3* KO cells complements the function of *Diaph3. Diaph3* KO cells expressing *Diaph1* and *Diaph2* (KO-*Diaph1*, KO-*Diaph2)* showed enlargement of the cells and an increase in the proportion of multinucleated cells at 39 °C (Figure 4D,E). In addition, the growth curve analysis indicated that KO-*Diaph1* and KO-*Diaph2* cells did not fully recover, unlike KO-*Diaph3* cells (Figure 4F,G). These results suggest that *Diaph3*, but not other formins, specifically restores the temperature sensitivity of *Diaph3* KO cells.

### 2.5. Diaph3 Regulates Cytokinesis via Controlling the Stability of Microtuble at High Temperatures

**Figure 5:**
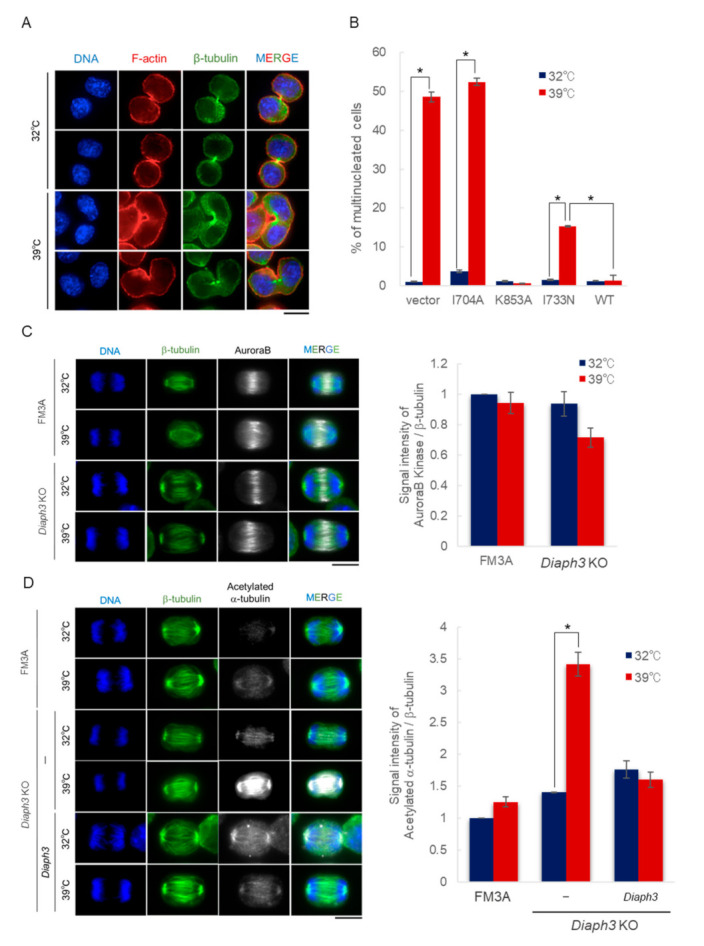
DIAPH3 contributes to the destabilization of microtubule in cytokinesis under high temperature conditions. (**A**) *Diaph3* KO cells were incubated at 32 and 39 °C for 24 h and immunostained with an antibody specific for β-tubulin (green). DAPI and Phalloidin were used to stain DNA (blue) and F-actin (red). The scale bar indicates 10 µm. (**B**) Control *Diaph3* KO cells (vector) and *Diaph3* KO cells that were expressing *Diaph3*^I704A^ (I704A), *Diaph3*^K853A^ (K853A), *Diaph3*^I733N^ (I733N), and *Diaph3* (WT) were cultured at 32 and 39 °C for 30 h. The cells were subjected to DAPI staining and photographed using a fluorescence microscope (BZ-X710). More than 300 cells were measured and the percentage of multinucleated cells was calculated; (error bars: ±SEM); * *p* < 0.001 (Dunnett’s test); 32 vs. 39 °C. (**C**) FM3A cells and *Diaph3* KO cells were incubated at 32 and 39 °C for 30 h and immunostained with antibodies specific for β-tubulin (green) and Aurora B (white). DAPI (blue) was used to stain chromosomes. The scale bar indicates 10 µm. Staining was examined by using a fluorescence microscope BZ710 with a microscope objective lense (CFI Plan-Apo 100×/1.45 NA, exposure time: 1/7.5 s). The signal intensity of Aurora B and β-tubulin on the equatorial plane (30 × 50 pixels) at anaphase was quantified using Image J, and the signal intensity of acetylated α-tubulin was normalized to that of β-tubulin. The bar graphs indicate the average of the normalized Aurora B intensity (right panel); (error bars: ±SEM) (Dunnett’s test); 32 vs.39 °C, *n* = 90. (**D**) FM3A cells and *Diaph3* KO cells were incubated at 32 and 39 °C for 30 h and then immunostained with antibodies specific for β-tubulin (green) and acetylated α-tubulin (white). DAPI (blue) was used to stain chromosomes. The scale bar indicates 10 µm. Staining was examined by using a fluorescence microscope BZ710 with a microscope objective lense (CFI Plan-Apo 100×/1.45 NA, exposure time: 1/6 s). The signal intensity of acetylated α-tubulin and β-tubulin on the equatorial plane (30 ×50 pixels) at anaphase was quantified using Image J, and the signal intensity of acetylated α-tubulin was normalized to that of β-tubulin. The bar graphs indicate the average of the normalized acetylated α-tubulin intensity (right panel); (error bars: ± SEM); * *p* < 0.001 (Dunnett’s test); 32 vs. 39 °C, *n* = 45.

We examined which stage was responsible for the cytokinetic abnormalities in *Diaph3* KO cells under high temperature conditions. Our results indicated that *Diaph3* KO cells formed the cleavage furrow and complete cytokinesis normally at 32 °C but showed abnormalities in the formation of the cleavage furrow at 39 °C (Figure 5A). The cleavage furrow is formed by the contractile ring composed of actin fibers, and DIAPH3 is known as the main factor responsible for actin fiber formation [17]. Therefore, it is possible that the contractile ring is disrupted due to the deletion of DIAPH3 at 39 °C, resulting in the failure of cytokinesis. Although DIAPH3 in tsFT101 cells has an I733N mutation on the FH2 domain, which is the actin nucleating domain, the mutant DIAPH3^I733N^ showed the same actin polymerization ability as WT, with no abnormalities in localization and reduced expression at 39 °C (Figures S7–S9). The I704A and K853A mutations on the FH2 domain of DIAPH3 have been reported to significantly reduce the actin polymerization ability [13]. Therefore, we expressed DIAPH3 mutants, I733N, I704A, and K853A, in *Diaph3* KO cells to test whether they recover from temperature sensitivity under high temperature conditions. As a result, only KO-DIAPH3^K853A^ cells recovered from temperature sensitivity at 39 °C (Figure 5B). These results suggest that the abnormalities in cell division under high temperature conditions occur regardless of the presence or absence of actin polymerization ability. At the same time, these results indicate that the main function of DIAPH3 under high temperature conditions might exist other than actin polymerization.

We hypothesized that *Diaph3* KO cells may fail in the accumulation of factors necessary for contractile ring formation in the equatorial plane at 39 °C. Microtubule dynamics play a major role in determining contractile ring formation and cleavage position in cytokinesis. In the late stages of cell division, contractile rings are formed around the equatorial plane due to the accumulation of factors necessary for the formation of contractile rings toward the plus end of the central spindle microtubules that are formed between sister chromatids distributed at both poles [18,19]. Aurora B binds to MKLP2, a kinesin-like protein, and is transported towards the plus end of the central spindle microtubule and accumulates in the cleavage plane [20]. Furthermore, it has been reported that cytokinetic defects are observed when the Aurora B activity is inhibited in late stages [21]. Therefore, we compared the localization of Aurora B at 32 and 39 °C in *Diaph3* KO cells. The results showed that there was no difference in the localization of Aurora B at 32 and 39 °C. Additionally, the fluorescence intensity of Aurora B in the equatorial plane tended to be lower at 39 °C, but there was no significant difference between 32 and 39 °C (Figure 5C).

Next, we examined whether DIAPH3 is involved in the stability of microtubules. Although formin family proteins are known as actin polymerization promoters highly conserved in eukaryotes, its microtubule-related functions have also been reported [22,23,24,25,26]. For example, *Daam* and *Inf2* are involved in microtubule acetylation, and *Diaph2*, an isoform of *Diaph3*, is involved in microtubule dynamics [25,27]. To test whether *Diaph3* also has microtubule-related functions, we focused on acetylated α-tubulin, a sign of stabilized microtubules, and observed its level. The results showed a significant increase in fluorescence intensity at 39 °C in *Diaph3* KO cells. Moreover, re-expression of *Diaph3* reduced the level of acetylated α-tubulin to the same level as FM3A cells (Figure 5D). Therefore, these results suggest that *Diaph3* regulates cytokinesis under high temperature conditions via decreasing the stability of microtubules by controlling the acetylation level of α-tubulin directly or indirectly.

## 3. Discussion

In this study, we investigated the cause of temperature sensitivity of mouse ts mutant strains to reveal a new mechanism of mammalian cell division. Not only tsFT50 and tsFT101 cells, for which *Diaph3* was suggested to be the gene responsible for temperature sensitivity, but also *Diaph3* KO cells showed temperature sensitivity. In addition, *Diaph3* KO cells that re-expressed *Diaph3* grew normally and recovered from temperature sensitivity even at restrictive temperatures. These results indicated that the requirement of *Diaph3* is dependent on temperature. *Diaph3* is one of the formin family members that play a role in actin polymerization [28,29,30,31,32]. In particular, *Diaph3* is thought to be an important factor responsible for the formation of the contractile ring in cytokinesis [17]. Watanabe et al. reported that the *Diaph3* knockdown by siRNA treatment causes the binucleation of cells and abnormal localization of contractile ring in mouse NIH3T3 cells at 37 °C [17]. The phenotypes are similar to our results obtained from *Diaph3* KO FM3A cells at 39 °C, and the report appears to support our finding in this study. Therefore, the actin polymerization ability was considered to be an essential function required at 39 °C. However, not all mutations on the FH2 domain of *Diaph3* are associated with multinucleation at 39 °C (Figure 5B). For example, DIAPH3^K853A^ rescued the multinucleation, but DIAPH3^I704A^ did not rescue, although both mutations are known to be important for the actin polymerization ability [13]. Therefore, only the defect in the actin nucleating function of the FH2 domain cannot explain the temperature sensitivity observed in *Diaph3* KO cells and ts mutants, and *Diaph3* might have some functions other than the actin polymerization ability. Although the FH2 domain is the actin nucleating domain, DIAPH3 has been reported to bind microtubules via its FH2 domain [29]. The difference between mutations on the FH2 domain may affect its interaction with actin and/or microtubule. It is possible that the disruption of the interaction might be severe in the case of I704A and lead to temperature sensitivity in *Diaph3* KO cells. 

Why is *Diaph3* necessary for cell division at 39 °C? We hypothesize that the stability of microtubules is a clue to answer this question. In this study, it is suggested that DIAPH3 contributes to microtubule instability under high temperature conditions. Microtubule stability is important in various cell division processes, including spindle formation, spindle checkpoint, and central spindle formation. β-tubulin tends to become unstable when exposed to low temperature environments [33,34,35], which may lead to differences in intracellular microtubule stability between the temperatures of 32 and 39 °C. Accordingly, we hypothesize that *Diaph3* may contribute to the tight control of the cell division mechanism by controlling the increased stability of microtubules under high temperature conditions. DIAPH1, DIAPH2, and DIAPH3 have been reported to directly bind to microtubules via the FH2 domain in vitro [29,36]. Therefore, DIAPH3 may contribute to the regulation of microtubule stability in cytokinesis through the binding of the FH2 domain to microtubules under high temperature conditions. In addition, based on the results of multinucleation analysis using the mutant DIAPH3 (Figure 5B), it is likely that I704 and I733 are involved in the destabilization of the microtubule. On the other hand, DIAPH2 has been reported to be involved in microtubule dynamics without the FH2 domain [27], and it is possible that DIAPH3 possesses functional domains for microtubule regulation other than the FH2 domain. The analysis of DIAPH3 domains by using deletion mutants will help elucidate the function of *Diaph3* in cytokinesis.

We suspect that the heat shock protein (*Hsp*) 72 is responsible for the difference in cell division mechanisms between 32 and 39 °C. The reasons are as follows: *Hsp72*, a HSP70 family protein, is induced by various stresses such as heat stress and plays a role in stabilization and folding of proteins. [37,38,39,40]. Heat stress affects the cell cycle by causing a variety of cell damages through thermal denaturation of proteins and through the production of reactive oxygen species [41,42,43,44,45]. It has been suggested that *Hsp72* is involved in the regulation of cell division under heat stress conditions. For example, *Hsp72* plays an important role in the completion of mitosis by protecting from heat stress-induced damage to centrosomes and mitotic abnormalities [43]. Additionally, *Hsp72* mobilizes the ch-TOG-TACC3 complex to promote a stable K-fiber assembly, thereby participating in the progression of cell division [46]. However, the functions of *Hsp72* are fully understood, and we wondered if other *Hsp72* functions may be involved in preventing mitotic abnormalities. In this study, we found that *Diaph3* is required for cell division only at 39 °C. Both *Diaph3* and *Hsp72*, which are required under high temperature conditions, may directly or indirectly function with each other to prevent cell division failure under heat stress.

The dysregulated expression of *Diaph3* has been observed in various cancer cells and is associated with cancer malignancy [47,48,49]. As mentioned above, *Diaph3* appears to be involved in the regulation of microtubule stability in cell division. Therefore, a more detailed analysis of the role of *Diaph3* in cell division will contribute to the elucidation of the function of *Diaph3* in microtubule stability and the mechanism underlying its effect on cancer malignancy, which may eventually lead to the application of *Diaph3* as a targeted therapy. In addition, cancer cells are more sensitive to temperature than normal cells and die at 40–44 °C because of low oxygen and pH states. Therefore, thermotherapy is widely used as a type of cancer treatment [50]. In this study, our results suggest that the requirement of *Diaph3* varies in a temperature-dependent manner. Consequently, low expression levels of *Diaph3* can be expected to result in failure of cell division and death at high temperatures, which may increase sensitivity to hyperthermia. In the future, we would like to elucidate the mechanism by which *Diaph3*-deficient cells die specifically at high temperatures and the correlation between the *Diaph3* expression level and cancerous growth of the cells. In addition, we expect to develop new therapeutic strategies for cancer treatment by controlling *Diaph3* expression levels. For this purpose, it is necessary to explore the function of *Diaph3* under high temperature environments. By elucidating unknown functions of *Diaph3* and its partner proteins related to the functions, we hope to clarify the mechanisms by which *Diaph3* is mobilized under high temperature environments.

## 4. Materials and Methods 

### 4.1. Cell Culture

FM3A cells, tsFT101 cells, and tsFT50 cells were cultured in RPMI1640 (with L-glutamine and phenol red) (FUJIFILM Wako Pure Chemical Corporation, Osaka, Japan) containing 10% adult bovine serum (CS) (Thermo Fisher Scientific, Carlsbad, CA, USA) and 1% penicillin/streptomycin (Abt) (FUJIFILM Wako) at 32 or 39 °C in an atmosphere of 5% CO_2_. HEK293T cells were cultured in DMEM (with L-glutamine and phenol red) (FUJIFILM Wako) containing 10% fetal bovine serum (FBS) (Biowest, Muaillé, France) and Abt at 37 °C under 5% CO_2_.

### 4.2. Multinucleation Analysis

Cells cultured for 24 h at 32 or 39 °C were collected and fixed with 4% paraformaldehyde (PFA) for 10 min at room temperature (RT). After fixation, the cells were washed twice with phosphate-buffered saline (PBS), and PBS was added to adjust the concentration to 5.0–7.0 × 10^6^ cells/mL. Cell suspension (15 µL, 0.5–1.0 × 10^6^ cells) was placed on a MAS-coated glass slide and dried at 37 °C for 10–15 min. After treatment with 0.5% Triton X-100 (in PBS), cells on the slide were treated for 20 min with 5% BSA in PBS containing 2 µg/mL DAPI and 0.1 nM Acti-Stain 555 phalloidin. A drop of mounting medium (Dako, Glostrup, Denmark) was added, a cover glass was placed, and it was sealed with nail polish. Multinucleated cells were analyzed using a fluorescence microscope (BZ-X710) (KEYENCE, Osaka, Japan). The number of cells was measured using ImageJ software.

### 4.3. Immunostaining

Up to the treatment with 0.5% Triton X-100 (in PBS), the same method as that used in the multinucleation analysis described above was performed. The cells were blocked with 5% BSA in PBS at room temperature for 1 h. After removing the blocking solution, the cells were treated with 2 µg/mL DAPI, 0.1 nM Acti-Stain 555 phalloidin, and 5% BSA in PBS containing a primary antibody at 4 °C overnight. Next, the slide was washed with PBS, a secondary antibody diluted in 5% BSA was added, and the cells were incubated at room temperature for 1 h. The slide was then washed with PBS. A drop of mounting medium (Dako, Glostrup, Denmark) was added, a cover glass was placed, and it was sealed with nail polish. Cells were analyzed using a confocal microscope (LSM 510 META) (ZEISS, Oberkochen, Germany) or a fluorescence microscope (BZ-X710) (KEYENCE, Osaka, Japan). The number of cells was measured using ImageJ software.

### 4.4. Exome Analysis

Genomic DNA from cells was purified using a GenElute Mammalian Genomic DNA Miniprep Kit (Sigma-Aldrich, St. Louis, MO, USA) according to the manufacturer’s protocol. Exome sequencing was performed by Macrogen Japan (Tokyo, Japan), a commercial service. Briefly, a DNA library was prepared using the SureSelectXT Library Prep Kit (Illumina, San Diego, CA, USA), and exome capture was carried out using the SureSelect XT Mouse All Exon Kit (Agilent, Santa Clara, CA, USA) according to manufacturers’ instructions, and exome-enriched DNA fragments were sequenced by the Illumina Hi-seq 4000 system (Illumina, San Diego, CA, USA)).

Quality control on raw reads was performed using FastQC. Paired-end reads were aligned to a mouse reference sequence mm9 using the Burrows-Wheeler Aligner (BWA). Multiple mapped read pairs with identical external coordinates were collapsed to remove potential PCR duplicates using the Picard MarkDuplicates. Local realignment and mapping quality score recalibration were performed using the Genome Analysis Toolkit (GATK). The Annovar tool was used for functional annotation of variants, exonic functions, and nonsynonymous variants, such as stop-gain single nucleotide variants, splicing, and frameshift indels. Known variants that were reported by using dbSNP128 were excluded. Among the resultant variants, we selected homo variants and used them for our study.

Nucleotide sequence data reported are available in the DDBJ Sequenced Read Archive under the accession numbers DRX238139, DRX238140, and DRX238141.

### 4.5. Cloning and Lentivirus Infection

Cells stably expressing 3×FLAG-tagged *Diaph1*, *Diaph2*, and *Diaph3* were established by lentiviral transduction. Briefly, lentivirus vector plasmids, psPAX2 (Addgene, Watertown, MA, USA), and pCMV-VSG-G (Addgene, Watertown, MA, USA) were transfected into HEK293T cells using Lipofectamine 3000 (Thermo Fisher Scientific, Waltham, MA, USA) according to the manufacturer’s instruction, and the medium was exchanged 16 h after the transfection. After incubation at 32 h, the supernatants were collected and added to cells together with 100× polybrene, followed by centrifugation (876× *g*, 90 min, 32 °C). The cells were resuspended in a RPMI1640 (with 10% CS and Abt) medium and cultured at 32 °C. Forty- eight to 72 h after virus infection, the cells were subjected to drug selection using blasticidin at a final concentration of 25 µg/mL. 

### 4.6. Western Blot (WB) Analysis

Cells were washed with PBS, lysed with 1% SDS in PBS, and then incubated at 95 °C for 5 min. Sonication (Handy Sonic UR-20P) (TOMY SEIKO, Tokyo, Japan) was performed, and the lysate obtained was diluted using an SDS sample buffer (0.25 M Tris-HCl pH 6.8 containing 50% glycerol, 10% SDS, 0.025% bromophenol blue (BPB), 0.5 M dithiothreitol (DTT)), and the mixture was heated at 95 °C for 5 min. Proteins were separated by the SDS-PAGE and transferred onto a PVDF membrane, followed by blocking TBS-T containing 5% skim milk. Next, the membrane was incubated with a primary antibody solution diluted with 5% skim milk in TBS-T (in 0.02% NaH_3_) at 4 °C overnight. After washing with TBS-T, the membrane reacted with a secondary antibody solution diluted in TBS-T containing 5% skim milk for 30 min. After washing with TBS-T, the signal was detected using ECL Western Blotting Detection Reagents (GE Healthcare, Chicago, IL, USA) with the imaging analyzer LAS4000 (GE Healthcare, Chicago, IL, USA). The antibodies used in Western blotting were as follows: Anti-β-tubulin (T4026) (Sigma-Aldrich, St. Louis, MO, USA), anti-acetylated α-tubulin (T7451) (Sigma-Aldrich, St. Louis, MO, USA), anti-γ-tubulin (ab179503) (Abcam, Cambridge, UK), anti-Aurora B (611082) (BD, San Jose, CA, USA).

### 4.7. Diaph3 Knockout by the CRISPR-Cas9 System

Forward (F) and reverse (R) oligo DNAs (5 µL containing 100 µM) shown below were mixed with 40 µL of 150 mM NaCl and incubated at 95 °C for 3 min, followed by incubation at 37 °C for 1 h. 

*Diaph3* CRISPR-1 F:5′-CACCGCTTCTCCCCGGGCTCGTAG-3′

*Diaph3* CRISPR-1 R: 5′-AAACCTACGAGCCCGGGGAGAAGC-3′ 

*Diaph3* CRISPR-2 F:5′-CACCGGGACAGCAAGTCGTCGCGG-3′

*Diaph3* CRISPR-2 R: 5′-AAACCCGCGACGACTTGCTGTCCC- 3′ 

This annealing solution was incubated with T4 PNK (37 °C, 30 min; 70 °C, 10 min). The annealed oligo DNAs were inserted into a psgRNA-AcGFP1 vector, which expresses sgRNA and AcGFP1 under the control of constitutive U6 and PGK promoters, respectively. 

FM3A cells stably expressing hSpCas9 (FM3A-hSpCas9) were established by lentiviral transduction. The FM3A-hSpCas9 cells were collected and washed twice with the Opti-MEM medium. Later, 1.3 × 10^7^ cells were suspended in Opti-MEM, and 25 µg of psgRNA-AcGFP1-*Diaph3* was added. The cell suspension was added to a cuvette, and electroporation was performed using the Super Electroporator NEPA21 (Nepagene, Chiba, Japan). The cells were cultured at 32 °C for 48 h and subjected to drug selection. The resultant cells were prepared at 5.0 × 10^6^ cells/mL, and a cell population with a strong fluorescent signal was fractionated using a cell sorter (SH800) (SONY, Tokyo, Japan). The sorted cells were cultured at 32 °C. The induction of mutation by genome editing was confirmed using a Guide-it^TM^ Mutation Detection Kit (TaKaRa, Tokyo, Japan). Knockout of *Diaph3* was confirmed by Sanger DNA sequencing. The primers used for the conformation of genome editing and DNA sequencing are shown below:

MutDet-*Diaph3*-1 F:5′- GGTTCCCTGCGGCTCAGATT-3′

MutDet-*Diaph3*-1 R:5′-AATCCCGCCTTGTCACTGGG-3′ 

MutDet-*Diaph3*-2 F:5′-GCTTTGGCATCCCCGGAAAA-3′

MutDet-*Diaph3*-2 R:5′-GCAGTGACTACACAACCCGC -3′ 

## 5. Conclusions

In this study, we revealed *Diaph3* as the gene responsible for the temperature sensitivity of tsFT101 and tsFT50 cells. Furthermore, we found that *Diaph3* is necessary for cell division under high temperature conditions even in mouse FM3A cells. Our results indicated that *Diaph3* might regulate cytokinesis via controlling the stability of the microtubule under high temperature conditions. Our study gives a new insight into the mechanisms by which regulatory factors of cell division function in a temperature-dependent manner.

## Figures and Tables

**Table 1:** Exome analysis of tsFT101 cells.

Conditions for Refinement	Number of Variants
Variants found in tsFT101 cells	73,807
Variants that cause amino acid substitutions in exonic regions	14,207
Variants that are not registered in the dbSNP	5254
Variants that exist in homo	1952
Variants that exist only in tsFT101 cells	85 (number of genes: 75)
Variants introduced into the genes involved in cytokinesis	4 (*diaphanous related formin 3* (*Diaph3*), *actinin alpha 2* (*Actn2*), *tubulin-specific chaperone E* (*Tbce*), *exocyst complex component 2* (*Exoc2*))

**Table 2:** Exome analysis of tsFT50 cells.

Conditions for Refinement	Number of Variants
Variants found in tsFT50 cells	1440
Variants that cause amino acid substitutions in exonic regions	558
Variants that are not registered in the dbSNP	534
Variants that exist in homo	37
Variants that exist only in tsFT50 cells	11
Variants introduced into the genes involved in cell division	4 (*diaphanous related formin 3* (*Diaph3*), *TAO kinase 1* (*Taok1*), *baculoviral IAP repeat containing 6* (*Birc6*), *dystrophin, muscular dystrophy* (*Dmd*))

## Supplementary Materials

The following are available online at https://www.mdpi.com/1422-0067/21/22/8493/s1.
