# Supplementary material for: Loss of DIAPH3, a Formin Family Protein, Leads to Cytokinetic Failure Only under High Temperature Conditions in Mouse FM3A Cells
Source: Int J Mol Sci. 2020 Nov 11;21(22):8493. doi: 10.3390/ijms21228493 (PMC7696919; doi:10.3390/ijms21228493)
Supplement: Supplementary file 1 [file ijms-21-08493-s001.pdf]

International Journal of
Molecular Sciences

MDPI

Supplemental Materials

Supplementary Materials and Methods

Supplementary Figure S1-S10

Supplementary Materials and Methods

Purification of GST-6 x His-tagged DIAPH3 and DIAPH3^{I733N}

FM3A cells stably expressing GST (Nter)-6xHis (Cter)-tagged DIAPH3 and -DIAPH3^{I733N} were established by lentiviral transduction. The cells were pelleted and suspended with 2.5 ml of Lysis buffer (20 mM HEPES-NaOH (pH7.5), 150 mM NaCl, 2.5 mM $MgCl_2$, 1% NP-40, 1 mM DTT, 1×protease inhibitor (nacalai tesque, Nakagyo-ku, Kyoto, Japan), 1×phosphatase inhibitor (nacalai tesque), 50 µg/mL RNaseA), followed by incubation at 4°C for 30 m. After centrifugation at 15,000 *g* for 15 m, the supernatant was mixed with 500 µl of Glutathione Sepharose 4B and rotated for 2 h. The mixture was transferred onto a column and washed with 2 ml of Wash buffer (20 mM HEPES-NaOH(pH7.5), 150 mM NaCl, 2.5 mM $MgCl_2$, 1% NP-40, 1 mM DTT, 1/20 × phosphatase inhibitor) 3 times, followed by wash with 2 ml of P5 buffer (-imidazole) (50 mM $NaHPO_4$, 300 mM NaCl, 10% glycerol, pH8.0). 1 ml of PreScission Protease Buffer (50 mM $NaHPO_4$, 300 mM NaCl, 10% glycerol, 80 units/ml PreScission Protease) was added to the column, and beads in the column was transferred to 1.5 ml tube and rotated at 4°C. The beads solution was transferred to the column and collected the elution. 500 µl of P5 buffer (-imidazole) was added onto the column twice, and imidazole was added to the elution (final 5 mM). The solution was mixed with 500 µl of Ni-NTA agarose and rotated for 1 h. After transferring onto a column, the mix was washed 3 times with 2 ml of P300 buffer (50 mM $NaHPO_4$, 300 mM NaCl, 10% glycerol, 30 mM imidazole, pH8.0). The column was washed with P500 buffer (50 mM $NaHPO_4$, 300 mM NaCl, 10% glycerol, 500 mM imidazole, pH8.0), and the elution was used as purified DIAPH3 proteins.

In vitro actin polymerization assay

3.93 M pyrene muscle actin (Cytoskeleton, Denver, CO, USA) was prepared by diluting with General Actin Buffer (2 mM Tri-HCl (pH8.0), 0.2 mM CaCl2, 0.2 mM ATP, 0.5 mM DTT), followed by 1h incubation at 4°C. After 2 h ultracentrifugation at 100,000 *g* at 4°C, the supernatant was collected and stored at 4°C. 1 µl of purified DIAPH3 or DIAPH3^{I733N} proteins was added to 49 µl of the resultant actin solution, and preincubated at 32°C or 39°C for 5 min. The solution was mixed with 50 µl of the Polymerization Buffer (500 mM KCl, 20 mM $MgCl_2$, 10 mM ATP), and the level of actin polymerization was measured by using RF-5300PC (SHIMADZU).

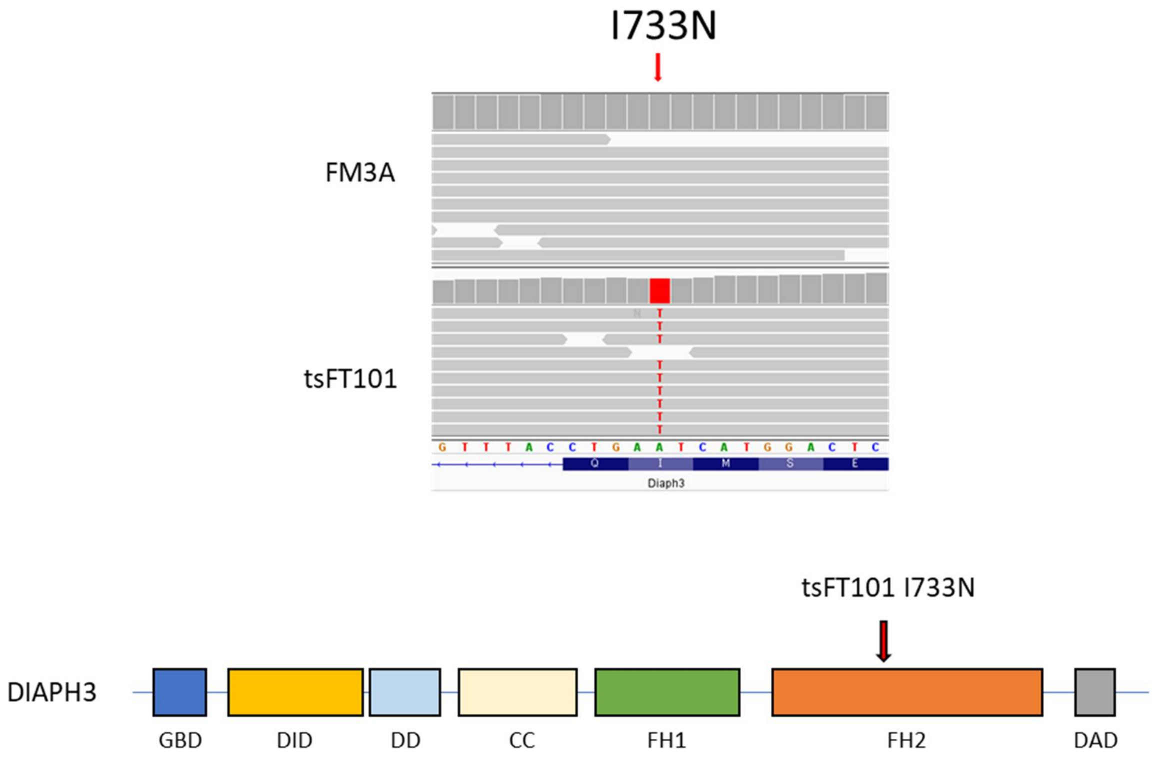

Figure S1. The *Diaph3* mutation present in tsFT101 cells.

GBD: GTPase-binding domain

DID: Diaphanous inhibitory domain

DD: Dimerization domain

CC: Coiled coil domain

FH1: Formin homology 1

FH2: Formin homology 2

DAD: C-terminal Diaphanous autoregulatory domain

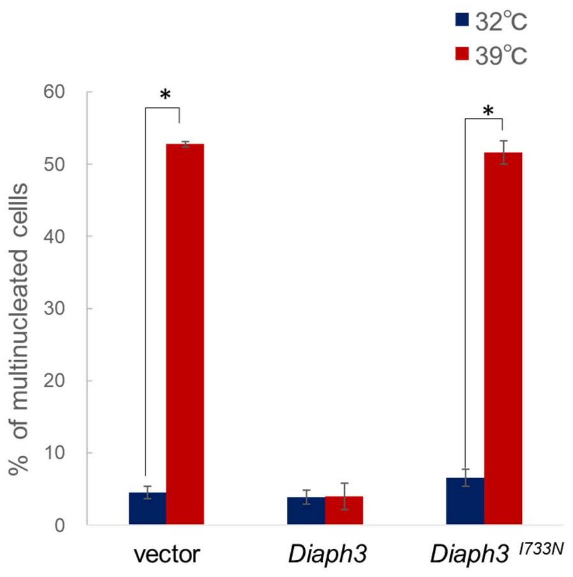

Figure S2. *Diaph3*^{I733N} did not recover multinucleation of tsFT101 cells. Control tsFT101 cells (vector), tsFT101 cells expressing *Diaph3* (*Diaph3*), and tsFT101 cells expressing *Diaph3*^{I733N} (*Diaph3*^{I733N}) were cultured at 32°C and 39°C for 24 h, stained with DAPI and photographed using a fluorescence microscope. More than 300 cells were measured and the percentage of multinucleated cells was calculated. (Error bar: ±SEM) *$p < 0.001$ (Student's t test); 32°C vs. 39°C

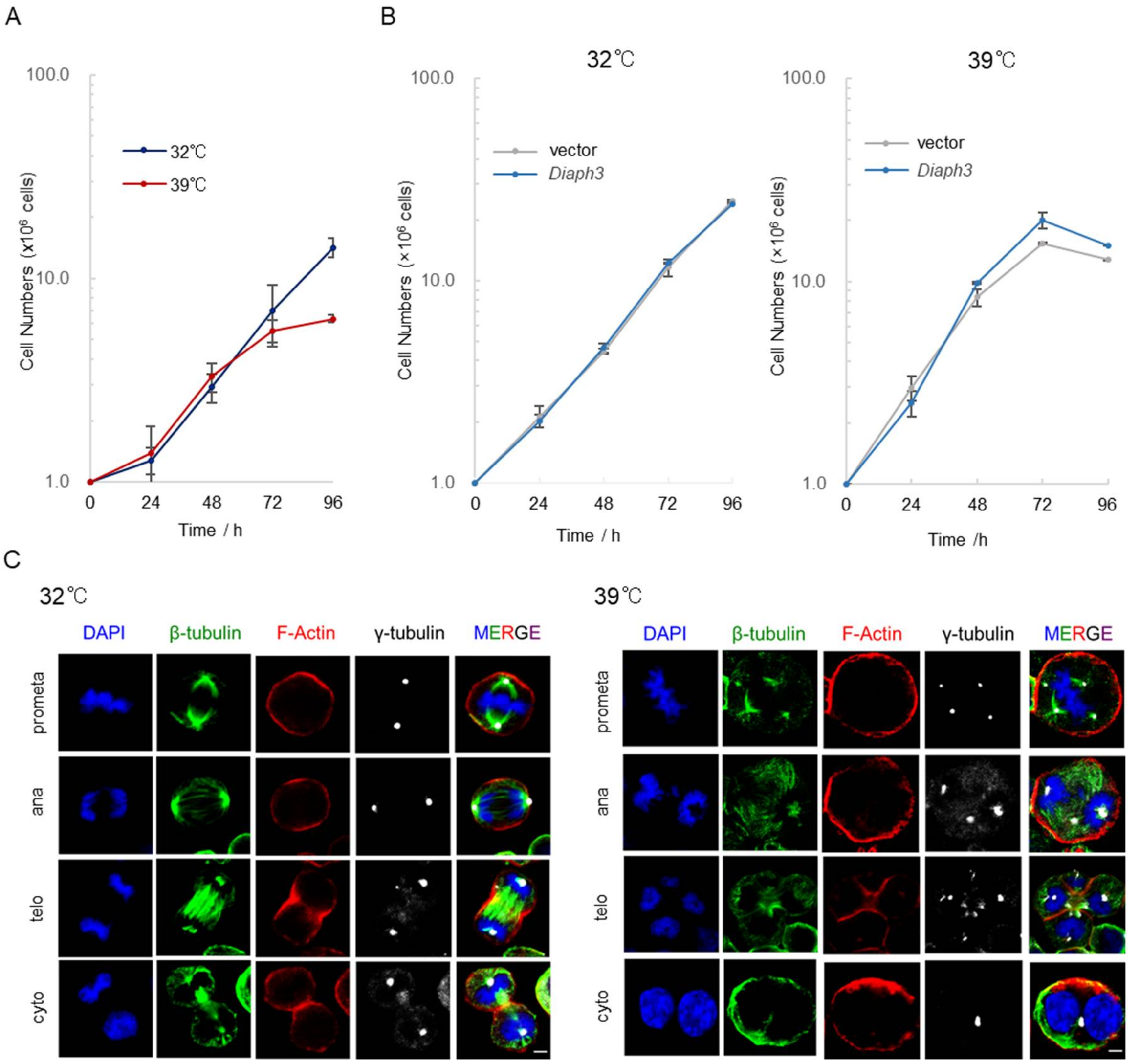

Figure S3. The phenotypes of tsFT50 cells and tsFT50-*Diaph3* cells. (A) Growth curve analysis of tsFT50 cells. Cells were seeded at 1.0 x 10^6 cells/dish and incubated at 32°C or 39°C, 5% CO_2, and collected every 24 h to measure the number of cells. (Error bar: ±SEM) *$p < 0.001$ (Student's t test); 32°C vs. 39°C (B) To observe the morphology of tsFT50 cells in cell division, they were incubated at 32°C and 39°C for 24 h and then immunostained with antibodies specific for β-tubulin and γ-tubulin. β-tubulin indicates microtubules (green) and γ-tubulin indicates centrosomes (white). DAPI (blue) and phalloidin (red) were used to stain chromosomes and F-actin, respectively. The scale bar indicates 10 μm. (C) Growth curve analysis of control tsFT50 cells (vector) and *Diaph3*-expressing tsFT50 cells (*Diaph3*). Cells were seeded at 1.0×10^6 cells/dish and incubated at 32°C or 39°C, 5% CO_2. The cells were collected every 24 h, and the number of cells was measured (Error bar: ±SEM). *$p < 0.001$ (Student's t test); vector (39°C, 96 h) vs. *Diaph3* (39°C, 96 h)

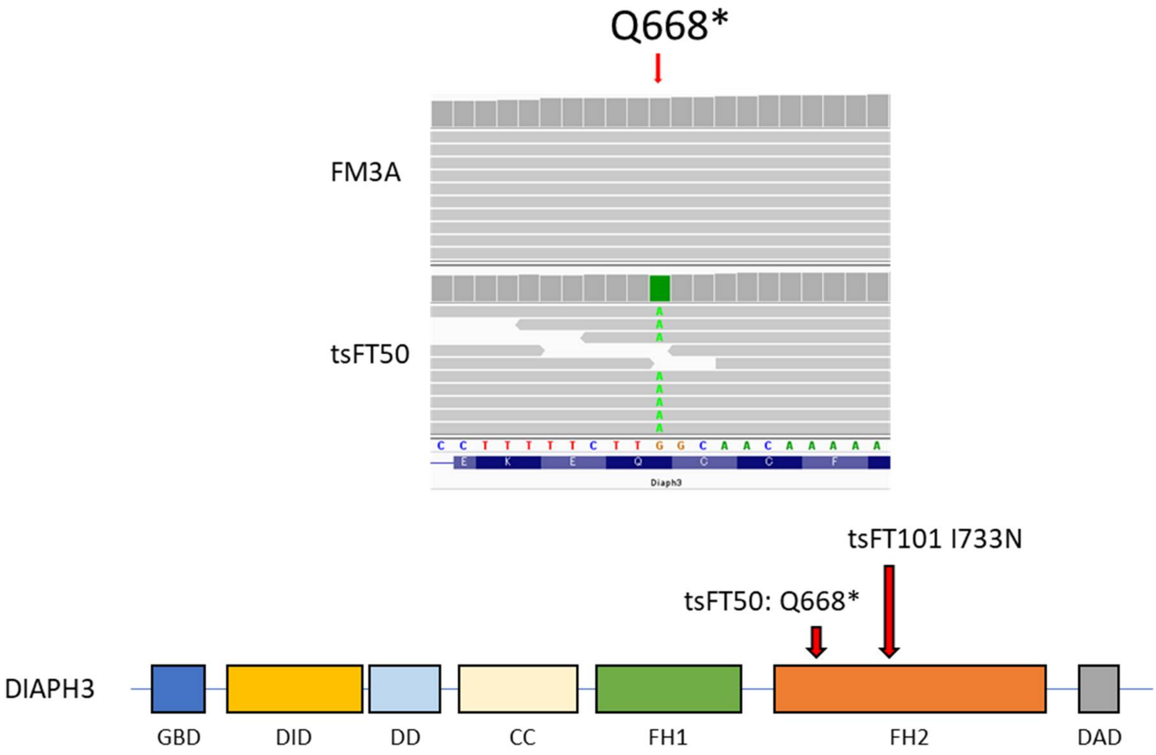

Figure S4. The *Diaph3* mutation present in tsFT50 cells.

GBD: GTPase-binding domain

DID: Diaphanous inhibitory domain

DD: Dimerization domain

CC: Coiled coil domain

FH1: Formin homology 1

FH2: Formin homology 2

DAD: C-terminal Diaphanous autoregulatory domain

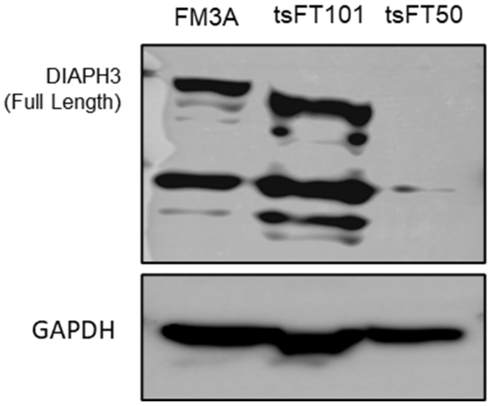

Figure S5. *Diaph3* expression in FM3A, tsFT101, and tsFT50 cells. Expression of *Diaph3* at 32°C in cells indicated was examined by W.B using an anti-DIAPH3 antibody. GAPDH was used as a loading control.

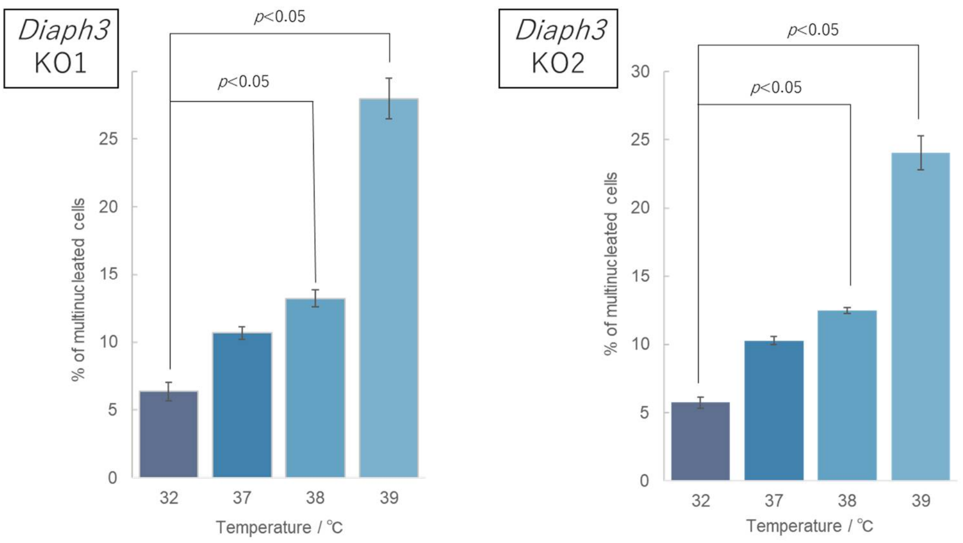

Figure S6. The multinucleation analysis of Diaph3 KO cells. *Diaph3* KO1 and KO2 cells were cultured at 32, 37, 38, and 39°C for 24 h, stained with DAPI, and photographed using a fluorescence microscope. More than 300 cells were measured, and the percentage of multinucleated cells was calculated. (Error bar: ±SEM)

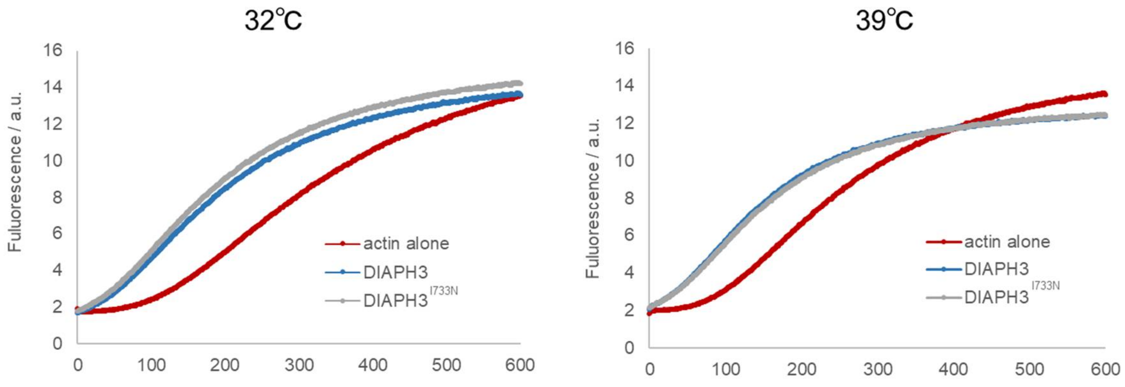

Figure S7. The actin polymerization activity of DIAPH3 and DIAPH3^{I733N}. In vitro actin polymerization assay was performed using purified DIAPH3 and DIAPH3^{I733N}. DIAPH3 proteins were reacted with pyrene actin at 32°C or 39°C, and the fluorescence level was measured.

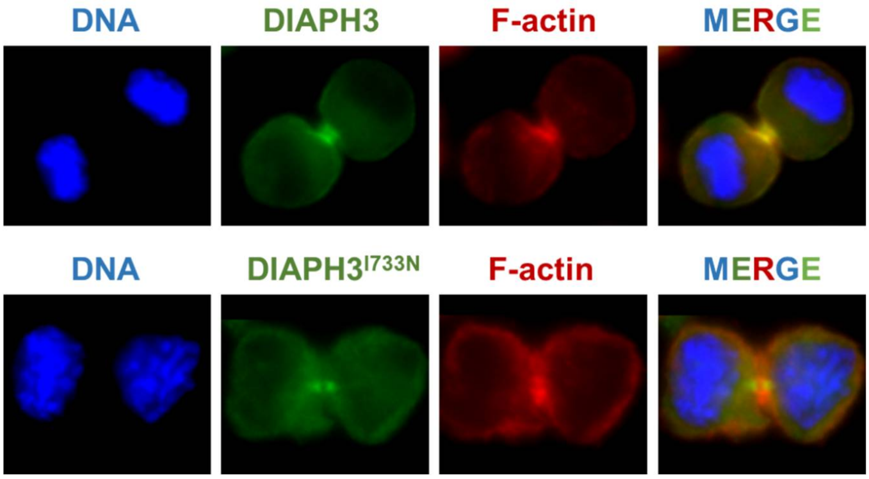

Figure S8. Localization of DIAPH3 and DIAPH3^{I733N}. GFP-tagged *Diaph3* and GFP-tagged *Diaph3^{I733N}* were expressed in FM3A cells. The localization of GFP-tagged DIAPH3 (top) and GFP-tagged DIAPH3^{I733N} (bottom) was analyzed. DAPI (blue) and phalloidin (red) were used to stain chromosomes and F-actin, respectively.

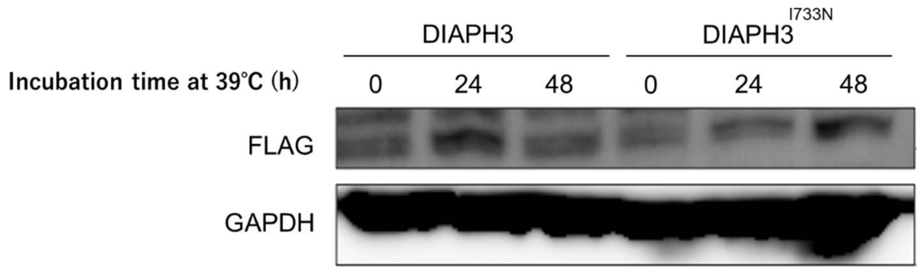

Figure S9. *Diaph3* and *Diaph3*^{I733N} expression in FM3A cells. FLAG-tagged *Diaph3* and FLAG-tagged *Diaph3*^{I733N} expressing FM3A cells were established, and the level of DIAPH3 and DIAPH3^{I733N} was examined by western blotting using an anti-FLAG antibody. GAPDH was used as a loading control.

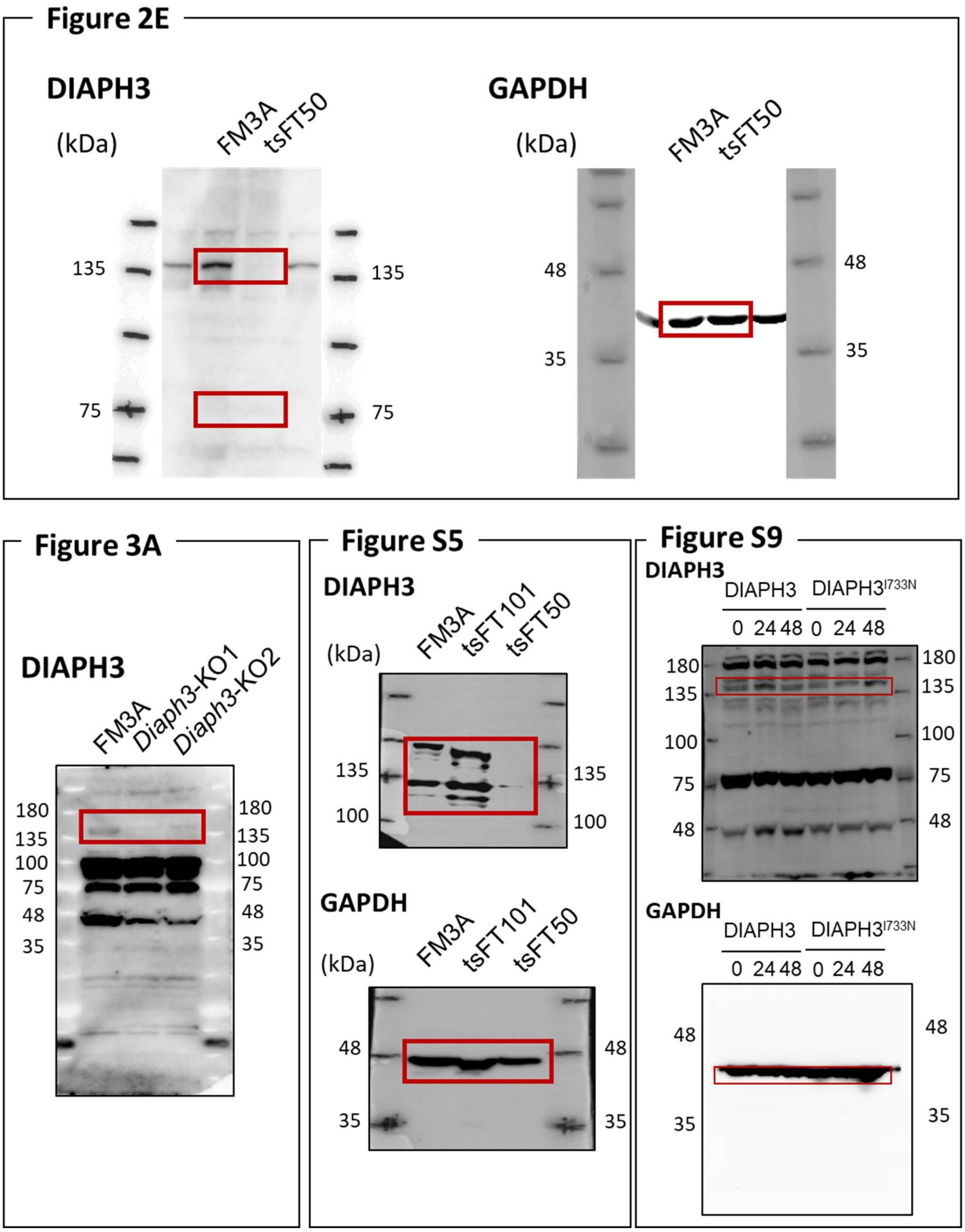

Figure S10. Original blot images. Blot images indicated by red boxes are used in Figures.
